# Indoor radon exposure and lung cancer: a review of ecological studies

**DOI:** 10.1186/s40557-016-0098-z

**Published:** 2016-03-25

**Authors:** Ji Young Yoon, Jung-Dong Lee, So Won Joo, Dae Ryong Kang

**Affiliations:** Department of Humanities and Social Medicine, Ajou University School of Medicine, Suwon, Korea; Office of Biostatistics, Ajou University School of Medicine, Suwon, Korea

**Keywords:** Radon, Lung cancer, Ecological study, Radon survey

## Abstract

Lung cancer has high mortality and incidence rates. The leading causes of lung cancer are smoking and radon exposure. Indeed, the World Health Organization (WHO) has categorized radon as a carcinogenic substance causing lung cancer. Radon is a natural, radioactive substance; it is an inert gas that mainly exists in soil or rock. The gas decays into radioactive particles called radon progeny that can enter the human body through breathing. Upon entering the body, these radioactive elements release α-rays that affect lung tissue, causing lung cancer upon long-term exposure thereto. Epidemiological studies first outlined a high correlation between the incidence rate of lung cancer and exposure to radon progeny among miners in Europe. Thereafter, data and research on radon exposure and lung cancer incidence in homes have continued to accumulate. Many international studies have reported increases in the risk ratio of lung cancer when indoor radon concentrations inside the home are high.

Although research into indoor radon concentrations and lung cancer incidence is actively conducted throughout North America and Europe, similar research is lacking in Korea. Recently, however, studies have begun to accumulate and report important data on indoor radon concentrations across the nation. In this study, we aimed to review domestic and foreign research into indoor radon concentrations and to outline correlations between indoor radon concentrations in homes and lung cancer incidence, as reported in ecological studies thereof.

Herein, we noted large differences in radon concentrations between and within individual countries. For Korea, we observed tremendous differences in indoor radon concentrations according to region and year of study, even within the same region. In correlation analysis, lung cancer incidence was not found to be higher in areas with high indoor radon concentrations in Korea.

Through our review, we identified a need to implement a greater variety of statistical analyses in research on indoor radon concentrations and lung cancer incidence. Also, we suggest that cohort research or patient-control group research into radon exposure and lung cancer incidence that considers smoking and other factors is warranted.

## Background

The International Agency for Research on Cancer reports that lung cancer incidence rates around the world are high [1]. Korea ranks third with 44.2 cases per 100,000 people. According to 2012 cancer registration statistics from the Korean Ministry of Health and Welfare, the incidence of lung cancer ranks fourth [2, 3], accounting for 9.9 % (22,118 people) of all cancers. Meanwhile, according to cause of death statistics [4] from the National Statistical Office of Korea, lung cancer has the highest mortality rate of 34.4 people per 100,000 people. The leading cause of lung cancer is smoking, followed by radon exposure. Radon, an inert gas, decays into radioactive particles called radon progeny that can enter the human body through breathing. Upon entering the body, these radioactive elements release α-rays that affect lung tissue, causing lung cancer upon long-term exposure thereto [5].

Previously, epidemiologic research found radon exposure to be a major cause of death among European miners. Beginning in the late 1980s, researchers throughout North America and Europe began to assess internal radon concentrations in homes. With the accumulated data, maps of radon-prone areas have been created, and studies thereof have begun to draw a link between indoor radon exposure and lung cancer incidence. Accordingly, the World Health Organization (WHO) has recommended regulations to reduce indoor radon concentrations, and has classified radon as a carcinogen, an agent that can cause cancer [6–8].

Notwithstanding, full-scale research into indoor radon concentrations in homes has only recently been undertaken in Korea, beginning in 2011. On a national scale, indoor radon concentrations in homes are measured in the winter every 2 years. Considering that the incidence rate and mortality rate of lung cancer in Korea are high, studies on whether or not domestic indoor radon concentrations affect lung cancer incidence are needed. Correlation research in this field, however, is lacking.

In the present study, we aimed to review indoor radon studies conducted in North America, Europe, and other countries in an attempt to better understand the characteristics of indoor radon concentrations in homes and to compare concentrations in these countries with those in Korea. We also attempted to outline correlations between indoor radon exposure and lung cancer incidence using the reviewed data. Lastly, we strove to highlight areas for future research that may help with understanding domestic radon concentrations, indoor radon exposure, and their effects on lung cancer incidence.

## Review

### A natural, radioactive element

Radon (radon-222) is a radioactive substance that is naturally produced during the radioactive decay of thorium and uranium, which are typically present in rocks. Radon is an inactive gas that is odorless and colorless, and it does not form chemical bonds with other substances. The half-life of radon is relatively long at 3.8 days, and it can stay in the air for a significant amount of time [9].

Radon emitted from soil into the air has a high dilution rate, and undergoes natural decay in the atmosphere. For this reason, radon concentrations are very low in outdoor environments. In an indoor environment that is not well-ventilated, however, radon concentrations may rise to levels that can have a damaging effect on the human body [10]. Source of radon present indoors can be traced to nearby soil and rocks, from which radon gas seeps into a building through cracks in its floors and walls, and to groundwater and construction materials brought into the building.

Nearly 85 % of the annual human radiation dose is attributable to natural radiation, and radon accounts for approximately 50 % of this radiation dose. Prolonged exposure to radon has been reported to increase the risk of lung cancer [11]. Radon exposure, however, is difficult to control and manage, as it is a naturally occurring radioactive substance. Nevertheless, national radon concentration surveys have been conducted across the world in order to set acceptable indoor radon levels as a means to protect people’s health [5, 12].

### Human influence of exposure to radon

Radon and its progeny, which exist in a particulate form, can stay in the air or be adsorbed to certain particles. Breathed into the human body, they adhere to the lung tissues. Emitted during the decay of radon and its progeny, α-rays have an impact on pulmonary cells, and can potentially cause lung cancer upon long-term exposure. Since even trace amounts of radon can have an impact on the human body, it is now considered a major carcinogen [13, 14]. The United States Environmental Protection Agency (USEPA) reported that 13.4 % of deaths resulting from lung cancer in 1995 were caused by radon exposure [15]. Meanwhile, the U.K., more than 1100 deaths resulting from lung cancer were reported as having been caused by radon exposure in homes [16].

Various factors must be considered when estimating the incidence of lung cancer caused by radon exposure. For this reason, epidemiological studies have been undertaken to assess radon levels and factors related to radon exposure in homes. Information directly related to residential radon concentrations has accumulated since the late 1980s [17]. Upon reviewing various data sources, the WHO reported that 3 to 15 % of all lung cancers worldwide were caused by radon exposure [5]. Comprehensive analyses of data from Europe, North America, and China have supported that the risk of lung cancer rises with an increase in cumulative radon exposure [18–20]. A comprehensive analysis of European data showed an increased risk of lung cancer for both smokers and non-smokers, while the relative risk per 100 Bq/m^3^ was found to be 1.11 (95 % CI 1.00–1.28) for those who have never smoked cigarettes in their lifetime [21]. A comprehensive study in North America showed a similar level of relative risk (1.10) for non-smokers, although it was not found to be statistically significant (95 % CI 0.91–1.42) [19]. These comprehensive analyses demonstrated radon in residential spaces to be a risk factor for lung cancer, even without cigarette smoking, which was consistent with results from the initial study into radon exposure conducted in European miners [19, 22].

According to the USEPA [9], in regards to lifelong exposure to radon at the action level, or recommended guideline of 4pCi/L, lung cancer is likely to develop in 63 out of every 1000 smokers and seven out of every 1000 non-smokers.

### Survey of indoor radon levels internationally

The USEPA, Public Health England, and the Radiation and Nuclear Safety Authority of Finland, among many other national institutions in North America and Europe, have performed national surveys to measure indoor radon concentrations and create maps of radon-prone areas, reflecting regional, geological differences in radon deposits (Table 1).

**Table 1:** Radon levels in homes throughout the world

Country	Period^[ref]^	Area	No. of dwellings	^222^Rn (Bq/m^3^)				
				AM(SD)	GM(SD)	Median (Min, Max)	Excessive rate (%)	Recommended level
USA	1989–1990 [12, 42]	Nationwide (sample size)	5,694	1.25(0.12)	0.68(0.08)	-	6.0	4pCi/L (USA unit is pCi/L)
	1991 [43]	Texas	2,890	1.0	0.5	0.6	3.6	
	2003–2004 [44]	Illinois	22,082	-	5.16(3.47)	3.6 (0.4, 178.9)	46.0	
	2010 [45]	Ohio	159,340	-	3.99	-(-, 927.6)	32.64	
	−2014 [46]	Kansas	73,959	5.1	-	-(-, 1,121.6)	42.6	
	−2014 [47]	New York	73,519	6.24	2.72(3.52)	-(-, 522.1)	-	
	−2015.6 [48]	Nevada	17,255	3.68	-	-	26.4	
Canada	2007 [49]	Ottawa	93	110(168)	74(2.26)	-(8,1525)	12.0	200
	2008 [50]	Winnipeg	116	143(101)	112(2.07)	-(20, 483)	20.0	
	2010 [51]	Fredericton	45	138(213)	82(2.56)	-(16, 1374)	18.0	
	2010 [51]	Halifax	64	259(475)	107(3.67)	-(4, 2341)	32.0	
UK	1986–1987 [52]	Nationwide (sample size)	2,093	20.5	15(2.2)	-	0.5	200
	−2009 [52]	England	465,000	99	53	-	11.3	
	−2009 [52]	Wales	16,800	91	51	-	10.7	
	−2008 [54]	Scotland	19,100	37	20	-(-, 4,600)	1.9	
	−2009 [55]	Northern Ireland	24,000	70	46	-(-, 4,900)	5.0	
Denmark	1985–1986 [56]	Nationwide	496	47	29(2.2)	-	2.2	200
	1995–1996 [57]	Nationwide	3,019	-	-	-(2, 590)		
	-[58]	Newly constructed	200	-	-	36.8 (9.0, 118)	7.0	100 (New homes)
Finland	1990–1991 [56]	Nationwide	3,074	123	84(2.1)	-	3.6 (Above 200 12.3)	400
	−1996 [59, 60]	Nationwide	51,443	248	-	-(-, 32,700)	13.0 (Above 200 33.0)	
Germany	1978–1984	Nationwide	7,500	50	40	-	1.5-2.5 (Above 200)	100
	1991–1993 [56]							
Hungary	1994–2006 [61]	Nationwide	6,154	174(139)	-	-(-, 1,841)	29.0	200
Ireland	1992–1999 [62]	Nationwide	11,319	89	57(2.40)	-(10, 1,924)	8.8	200
Japan	1993–1996 [63]	Nationwide	899	15.5(13.5)	12.7(1.78)	11.7 (-, 208)	-	-
	-[64]	Hokkaido, Hiroshima, Kochi	6,645	21.3(18.8)	17.3(1.83)	16.4 (-)	0.4 (Above 148)	
China	1984–1990 [65]	Nationwide	10,811	22.5	19.6	-	-	
	1994–1998 [66]	Shenyang	608	115.7	91.2(1.93)	122.4 (-)	17.4 (Above 148)	
		Gansu	2,394	222.9	176.2(2.08)	227.8	65.7 (Above 148)	

*AM* arithmetic mean, *SD* standard deviation, *GM* geometric mean, *Min* minimum, *Max* maximum

In the US, a nationwide indoor radon survey was conducted from 1989 to 1991 to create a map of radon-prone areas and to raise public awareness of the risks and countermeasures associated with radon: similar maps have been drawn by the USEPA to reflect indoor radon measurements, geology, aerial radioactivity, soil permeability, and foundation type [8]. The national radon concentration survey in the US involved sampling the indoor air of some 5600 homes nationwide for measurements of radon concentrations between 1989 and 1990. The results revealed a mean indoor radon concentration of 1.25 pCi/L, with 6.0 % of the homes having indoor radon concentrations exceeding the action level of 4pCi/L. The State of New York measured indoor radon concentrations for some 73,000 homes until 2014, and reported a mean indoor radon concentration of 6.2 pCi/L. In the State of Nevada, indoor radon concentrations for about 17,000 homes were measured until June 2015, and their results uncovered a mean indoor radon concentration of 3.7 pCi/L, with the radon concentrations of 26.4 % of homes exceeding the action level of 4 pCi/L.

Canada has also conducted regional surveys of indoor radon concentrations. In the latest indoor radon concentration survey, the mean indoor radon concentrations for Ottawa, Winnipeg, and Halifax were fairly high, at over 100 Bq/m^3^. Of particular note, in Halifax, 64 homes had a mean indoor radon concentration of 259 Bq/m^3^, and 32.0 % of the homes exceeded the action level of 200 Bq/m^3^ recommended by the Canadian government.

The UK introduced a radon reduction measure in 1987, and has set out to annually record mean radon concentrations in homes. The Health Protection Agency of the UK has created maps that indicate areas of high amounts of radon, which are divided into classes, based on indoor radon concentration data obtained from about 5.1 million homes in England, Wales, Scotland, and Northern Ireland. The first class is designated for areas with a 1 % or higher chance of a home having radon concentrations above the action level of 200 Bq/m^3^. In England, an indoor radon concentration survey conducted on about 460,000 homes until 2009 reported a mean indoor radon concentration of 99 Bq/m^3^, and 11.3 % of the homes had radon concentrations exceeding the action level of 200 Bq/m^3^. In Wales, however, the mean indoor radon concentration for 16,800 homes was 91 Bq/m^3^, with 10.7 % of the homes having radon concentrations that exceeded the action level.

In Denmark, national surveys on radon concentrations in houses were conducted in 1986 and 1996; the scope of the survey also included new housing. Their results indicated that 7 % of new houses had radon levels exceeding the acceptable level of 100 Bq/m^3^. In Finland, an indoor radon concentration survey has been conducted on 113,000 homes thus far, and high radon concentrations have been observed in the southern region near Helsinki [22].

In Germany, two indoor radon concentration surveys were conducted on a total of 7500 homes between 1978 and 1984 and 1991 and 1993. In Hungary, an indoor radon survey conducted on 6154 homes nationwide from 1994 to 2006 showed a mean radon concentration of 174 Bq/m^3^, with 29.0 % of the homes surpassing the recommended radon level of 200 Bq/m^3^. In Ireland, an indoor radon survey was conducted on more than 110,000 homes nationwide from 1992 to 1999, and the results revealed a mean indoor radon concentration of 89 Bq/m^3^, with 8.8 % of the homes having radon concentrations exceeding the recommended radon level.

In Japan, indoor radon concentrations for 899 homes nationwide were investigated from 1993 to 1996. Also, a similar survey was conducted on some 6600 homes in Hokkaido, Hiroshima, and Kochi. Radon levels were assessed in accordance with the USEPA action level of 148 Bq/m^3^, since a regulation standard for indoor radon concentrations has not yet been recommended for Japan, and the studies found that radon concentrations in 0.4 % of the homes exceeded the action level. In China, an indoor radon concentration survey was conducted on more than 100,000 homes nationwide from 1984 to 1990, and additional surveys were conducted in Gansu and Shenyang, where indoor radon concentrations are reportedly high.

### Nationwide survey of indoor radon levels in Korea

The International Commission on Radiological Protection Publication (ICRP)-103, issued in 2007, proposed the need for an efficient measure to minimize radon exposure that defines standards for indoor radon concentrations and designates areas at high risk for radon exposure, as part of a concentrated effort to manage indoor radon levels [23]. As natural radiation sources reflect the geological and geographical characteristics of a particular region and are closely associated with development and industrialization of a country, sufficient data accumulation for individual countries is essential to proper management of natural radiation sources, such as radon [24].

In Korea, radon concentration surveys were initiated in the 1980s by independent researchers to investigate indoor air pollution levels and measure radon gas concentrations. Since then, radon concentration surveys have been conducted by both independent researchers and national agencies, although at an inadequate level, compared to other nations. In this study, several radon concentration surveys conducted by national agencies in Korea were examined (Table 2).

**Table 2:** Summary of indoor radon concentrations in homes throughout Korea

no	Institution^[ref]^	Period	Area	No. of dwellings	^222^Rn (Bq/m^3^)			
					AM(SD)	GM(SD)	Median (Min, Max)	Excessive rate (ER)^a^ (%)
1	KINS [25]	1988 (winter)	7 cities^c^	530	99.9(-)	-	88.8(-)	16.0
2	KAERI [26]	1990.4–1990.10	Nationwide	340	59.57(-)	-	48.90 (-)	-
3	NIH [27–29]	1993	Nationwide	34	27.75(4.07)	-	-(8.14, 99.9)	-
4								
5		1994.9–12	Seoul	410	18.9(10.7)	-	19.70 (9.3, 30.7)	-
6		1995.9–12	Northern area of Gyeonggi	197	34.6(4.1)	-	-(18.5, 54.0)	-
7	KFDA [30, 31]	1996.9–11	Southern area of Gyeonggi	384	29.4(19.4)	-	-(7.4, 132.8)	-
8		1997.8–11	Incheon, Daejeon, Chungnam, Chungbuk	590	Incheon: 8.0(10.9)	-	Incheon: (2.7, 89.7)	-
					Daejeon: 25.0(12.6)		Daejeon: (5.7, 82.2)	
					Chungnam: 32.1(21.5)		Chungnam: (4.9, 145.7)	
					Chungbuk: 20.0(20.3)		Chungbuk: (2.7, 131.6)	
9	KINS [67]	1999.12–2000.11	Nationwide	2,190	53.4(57.5)	43.3(1.8)	39.8 (13.6, 1,350)	1.7 (200^b^)
10	KINS [33]	2002–2004	Nationwide	450	40.4(56.0)	10.7(2.9)	25.4 (-, 731)	-
11	NIER [34]	2011.12–2012.5	Nationwide	7,885	124.9(144.7)	91.2(2.1)	85.7 (7.0, 2,821.3)	22.2
12	NIER [35]	2013.11–2014.4	Nationwide	6,648	102.0(114.2)	74.9(2.0)	68.0 (9.8, 1,936.6)	16.3
			‘12 highest radon level A area	1,737	155.0(167.8)	107.4(1.1)	99.3 (7.4, 1,956.5)	32.1
13	CRIPHE [32]	2013.11–2014.3	Chungnam 5 province	114	92.5(-)	-	-(9.6, 640.4)	15.8

^a^ER is 148 Bq/m^3^ of the US Environmental Protection Agency’s (USEPA) recommended action level; ^b^200 Bq/m^3^ of the recommended value in International Commission on Radiological Protection (ICRP) 65; ^c^seven cities include Seoul, Chuncheon, Daejeon, Daegu, Gwangju, Pusan, and Jeju.

*AM* arithmetic mean, *SD* standard deviation, *GM* geometric mean, *Min* minimum, *Max* maximum, *KINS* Korea institute of nuclear safety, *KAERI* Korea atomic energy research institute, *NIH* national institute of health, *KFDA* Korea food and drug safety, *NIER* national institute of environmental research, *CRIPHE* Chungnam research institute public health and environment

The Korea Institute of Nuclear Safety (KINS) measured radon levels in homes in seven cities, including Seoul, Chuncheon, Daejeon, Daegu, Gwangju, Busan, and Jeju, over a 3-month period in the winter of 1988. Their results after analyzing radon levels for 530 homes showed that 16.0 % of the examined homes had radon concentrations exceeding 148 Bq/m^3^ [25]. The Korea Atomic Energy Research Institute examined indoor radon levels for 340 homes in 12 regions throughout the country from April to October 1990. The mean radon concentration recorded in their study was 59.6 Bq/m^3^ [26].

The National Institute of Health (NIH, presently the Center for Disease Control and Prevention) investigated indoor radon concentrations in Seoul and the Northern Gyeonggi-do Province from 1992 to 1995 in order to obtain basic data with which to assess the potential hazard associated with radon and its decay products [27–29]. In the first year (1992), the basements and first floors of 22 public health center buildings in Seoul were examined, and in the second year (1993), 34 residential facilities, including apartments, multiplex houses, row houses, and detached houses, were examined. In the third year (1994), the indoor radon concentrations of 410 homes in 22 districts of Seoul were examined; the mean radon concentration was 18.9 ± 10.7 Bq/m^3^. In the fourth year (1995), 197 homes in ten cities and counties in the Northern Gyeonggi-do Province were examined, and the mean indoor radon concentration was 34.6 ± 4.17 Bq/m^3^.

Following the NIH surveys in Seoul and the Northern Gyeonggi-do Province, the Korea Food and Drug Administration (presently the Ministry of Food and Drug Safety) measured indoor radon levels in homes within the Southern Gyeonggi-do Province, Incheon, and Chungcheong in 1996 and 1997. The indoor radon levels of 384 residential facilities in 21 cities and counties throughout the Southern Gyeonggi-do Province were measured between September and November of 1996, and 590 residential facilities in 38 cities, counties, and districts in Incheon, Daejeon, and Chungcheong-do Province were examined between August and November of 1997. The mean indoor radon concentration in homes was 29.4 Bq/m^3^ in the Southern Gyeonggi-do Province, and 8.0, 25.0, 32.1, and 20.0 Bq/m^3^ in Incheon, Daejeon, Chungcheongnam-do, and Chungcheongbuk-do, respectively [30, 31].

From December 1999 to November 2000 (four measurements), the KINS surveyed indoor radon levels in 3000 homes and public buildings in six metropolitan cities and nine provincial regions. Initially, 2500 homes nationwide were examined, although the data on only 2190 homes were included in the final analysis. The results of the survey showed a mean indoor radon concentration of 53.4 Bq/m^3^, and 1.7 % of the examined homes had indoor radon concentrations exceeding the ICRP-65 radon action level of 200 Bq/m^3^ [32]. From 2002 to 2004, 450 homes in seven metropolitan cities and nine provincial regions were examined, showing a mean radon concentration of 40.4 Bq/m^3^ [33].

In subsequent years, the National Institute of Environmental Research decided to conduct nationwide surveys of indoor radon levels in the winter every two years and to examine as many homes as possible in a short period of time. From December 2011 to May 2012, 7885 homes nationwide were examined to assess indoor radon concentrations and to identify environmental factors influencing indoor radon concentrations. The measurement results revealed a mean radon concentration of 124.9 Bq/m^3^, and 22.2 % (1752) of the homes examined had radon concentrations exceeding 148 Bq/m^3^, the acceptable indoor radon level recommended for multi-use facilities by the Ministry of Environment [34].

In the winters of 2013 and 2014, 6648 homes (52 % detached houses, 24 % multi-unit and row houses, and 24 % apartments) were examined in a survey of indoor radon levels. The mean radon concentration reached 102.0 Bq/m^3^, with 16.3 % of homes having indoor radon concentrations exceeding the recommended level [35]. Additionally, assessment of the environmental impact of soil and groundwater on radon levels was conducted in select regions, where indoor radon concentrations were found to be high in the 2011–2012 survey. Homes in these areas exhibited a mean radon concentration of 155.0 Bq/m^3^, and 32.1 % of the homes had indoor radon concentrations higher than 148 Bq/m^3^ [35].

The Chungnam Research Institute Public Health and Environment selected 114 homes across the Chungcheongnam-do Province, where high radon concentrations were observed in the national indoor radon survey in 2012. The mean radon concentration was 92.5 Bq/m^3^, with 15.8 % of the homes having indoor radon concentrations of over 148 Bq/m^3^ [32].

According to indoor radon concentration research in Korea, places with high indoor radon concentrations include Kangwondo, Choongcheondo, Jeonrado, and Kyoungsangbookdo. These areas of higher indoor radon concentration mostly coincide with geological distributions of radium deposits in granite and surface soil [34, 36, 37]. A similar link between indoor radon concentrations and geological deposits was also noted in North America and Europe. Accordingly, the Korean Ministry of Environment has since sought to inform residents of radon concentrations in each region through the Ministry of Environment Living Environment Information Center [38] via radon maps based on national house radon concentrations reported by the NIER [34, 35].

In the present review, when we compared radon maps for each year in Korea, we noted large differences in radon concentrations according to region and year of study, even within the same region. This, however, was not limited to only within Korea: The United Nations Scientific Committee on the Effects of Atomic Radiation has been collecting, organizing, and announcing indoor radon concentrations for all countries. In their data, we identified marked changes in radon concentrations for each country [39].

### Indoor radon exposure and lung cancer

To investigate correlations between indoor radon exposure and lung cancer in Korea, we drew and compared graphs of indoor radon concentration data for 7885 homes across the country, as reported by the NIER [34], and lung cancer incidence data [40] for 2011–2013. We only used data for women, who are believed to be less affected by smoking, to control for smoking, which is the leading cause of lung cancer. Data are listed in order of administrative district with the highest indoor radon concentrations to that with the lowest.

**Fig. 1:**
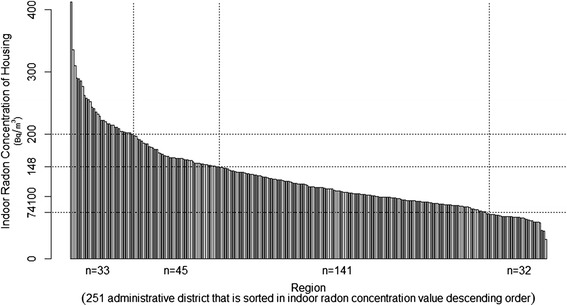
Mean indoor radon concentrations in homes throughout 251 administrative districts in Korea (December 2011 - May 2012). Source: National Institute of Environmental Research in Korea. 1.Hwacheon-gun(562), 2.Jinan-gun(412), 3.Yeongwol-gun(335), 4.Jangsu-gun(310), 5.Muju-gun(290), 6.Goseong-gun(288), 7.Hoengseong-gun(285), 8.Pocheon-si(277), 9.Taebaek-si(262), 10.Damyang-gun(258), 11.Hongcheon-gun(255), 12.Wanju_Gun(252), 13.Goheung-gun(244), 14.Inje-gun(241), 15.Cheongsong-gun(235), 16.Geumsan-gun(232), 17.Sunchang-gun(229), 18.Danyang-gun(222), 19.Boeun-gun(222), 20.Nonsan-si(220), 21.Jeongseon-gun(217), 22.Yeongam-gun(216), 23.Mungyeong_si(214), 24.Pyeongchang-gun(214), 25.Gapyeong-gun(211), 26.Sokcho-si(211),…, 247.Seoul Seongdong-gu(58), 248.Inchen Dong-gu(57), 249.Ulsan Nam-gu(46), 250.Ulsan Jung-gu(44). 251.Changwon Seongsan-gu(31)

**Fig. 2:**
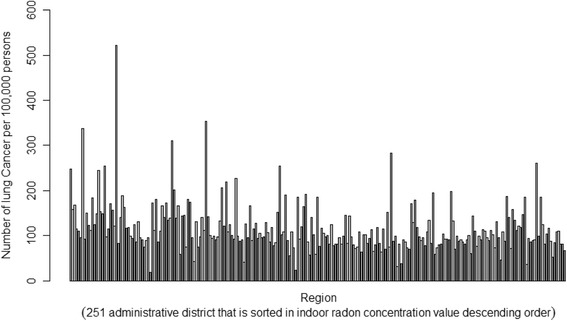
Number of lung cancer patients per 100,000 persons (females, 2011-2013) throughout 251 administrative districts in Korea. Source: National Health Insurance Corporation in Korea. 1.Hwacheon-gun(562), 2.Jinan-gun(412), 3.Yeongwol-gun(335), 4.Jangsu-gun(310), 5.Muju-gun(290), 6.Goseong-gun(288), 7.Hoengseong-gun(285), 8.Pocheon-si(277), 9.Taebaek-si(262), 10.Damyang-gun(258), 11.Hongcheon-gun(255), 12.Wanju_Gun(252), 13.Goheung-gun(244), 14.Inje-gun(241), 15.Cheongsong-gun(235), 16.Geumsan-gun(232), 17.Sunchang-gun(229), 18.Danyang-gun(222), 19.Boeun-gun(222), 20.Nonsan-si(220), 21.Jeongseon-gun(217), 22.Yeongam-gun(216), 23.Mungyeong_si(214), 24.Pyeongchang-gun(214), 25.Gapyeong-gun(211), 26.Sokcho-si(211),…, 247.Seoul Seongdong-gu(58), 248.Inchen Dong-gu(57), 249.Ulsan Nam-gu(46), 250.Ulsan Jung-gu(44). 251.Changwon Seongsan-gu(31)

Comparing the graphs, we determined that lung cancer incidence was not higher in areas with higher indoor radon concentrations. Indeed, lung cancer incidence was similar for all areas (Figs. 1 and 2). Globally, however, results differ. Studies in other parts of the world suggest an association between indoor radon exposure and lung cancer. Nevertheless, in a KINS study [41], a correlation between indoor radon concentrations and lung cancer incidence in Korean males was reported; however, there was no correlation between indoor radon concentrations and lung cancer incidence in females or lung cancer deaths in either sex.

Expounding on our results would be difficult, as our analysis was limited by not being able to control for lifestyles or habits, history of disease, socioeconomic status, etc. Also, as we simply compared indoor radon concentrations and lung cancer incidence data, it would be beyond the scope of the study to draw a correlation between indoor radon concentration and lung cancer with our results alone. Accordingly, we suggest a need for further research into the effects of indoor radon concentrations on lung cancer incidence that employs a variety of statistical methods. Suggesting average values by clustering areas in which large differences in indoor radon concentrations are recorded may produce useful data for future research.

## Conclusion

Radon is a carcinogenic substance and the second leading cause of lung cancer after smoking. As Korea has high incidence and mortality rates for lung cancer, a link between radon exposure and lung cancer may be present, although one was not observed in the present study. Nevertheless, research thereon has mostly been conducted by individual, regional study groups in Korea. Moreover, within the available data, there is a possibility of ecological errors from not being able to control for uncertainty in individual lifestyles or habits, history of disease, socioeconomic status, etc. Accordingly, we suggest a need for ecological research that applies more specialized statistical analyses and cohort research or patient-control group research that seeks to control individual differences in smoking, radon exposure, etc.
